# Histological, Cytological, and Radiological Correlation in Thyroid Lesions

**DOI:** 10.7759/cureus.98286

**Published:** 2025-12-02

**Authors:** Zainab Adil, Rajni Choudhary, Maneesh Sulya, Mahim Koshariya, Monalisa Tekam

**Affiliations:** 1 Department of Pathology, Gandhi Medical College, Bhopal, IND; 2 Department of General Surgery, Gandhi Medical College, Bhopal, IND

**Keywords:** cytology-histology correlation, diagnostic accuracy, fine-needle aspiration cytology, histopathology, thyroid lesions, tirads

## Abstract

Background: Thyroid disorders are among the most common endocrine diseases, with lesions ranging from benign nodules to malignant neoplasms. Accurate preoperative diagnosis is crucial for appropriate management. Fine-needle aspiration cytology (FNAC) is a widely used diagnostic tool, while ultrasonography (USG) and histopathology provide valuable adjunctive information. Correlating these modalities enhances diagnostic accuracy and clinical decision-making.

Objectives: To evaluate the cytological, histopathological, and radiological correlation in thyroid lesions and determine the diagnostic accuracy of FNAC and Thyroid Imaging Reporting and Data System (TIRADS) in differentiating benign and malignant thyroid nodules.

Methodology: This cross-sectional, observational study was conducted in the Department of Pathology at Gandhi Medical College and Associated Hospitals, Bhopal, from May 2023 to October 2024, following approval of the ethics committee. A total of 150 samples from patients presenting with thyroid swellings were included in the study. All patients underwent clinical evaluation, thyroid function testing, USG (classified using the TIRADS system), and FNAC. Surgical specimens were subjected to histopathological examination wherever available.

Results: The majority of patients were middle-aged females, with a female-to-male ratio of 4.6:1. The majority of lesions were benign, with colloid goiter being the most common diagnosis (40, 26.7%), followed by lymphocytic thyroiditis (17, 11.3%) and nodular goiter (15, 10%). On USG, most nodules were TIRADS 2 (58, 38.7%) and TIRADS 3 (63, 42%), while all TIRADS 5 lesions were malignant on histopathology (*P *< 0.001). FNAC demonstrated a diagnostic adequacy of 96%, with an overall sensitivity of 62.5%, a specificity of 97.7%, and a diagnostic accuracy of 92.3%. Strong cytoradiological and cytohistological concordance was observed in benign and malignant categories.

Conclusions: The integration of cytological, radiological, and histopathological evaluation significantly improves the diagnostic precision of thyroid lesions. FNAC remains a simple, cost-effective, and reliable first-line investigation, while TIRADS scoring effectively stratifies the risk of malignancy. The combined use of these modalities facilitates accurate diagnosis, rational surgical intervention, and optimal patient management.

## Introduction

The thyroid gland is a highly vascular endocrine organ located in the anterior neck, playing a crucial role in metabolic regulation, growth, and development by secreting the thyroid hormones thyroxine (T4) and triiodothyronine (T3) [1]. Thyroid lesions encompass a broad spectrum of disorders ranging from benign colloid nodules and thyroiditis to malignant neoplasms such as papillary, follicular, medullary, and anaplastic carcinoma [2]. The global incidence of thyroid nodules is estimated at 4%-7% clinically and up to 67% when detected by ultrasonography (USG), with a higher prevalence among females and elderly individuals [3]. Although most nodules are benign, approximately 5%-10% harbor malignancy, underscoring the importance of accurate diagnosis [4].

Fine-needle aspiration cytology (FNAC) is the cornerstone of thyroid lesion evaluation due to its simplicity, cost-effectiveness, and high diagnostic accuracy. It allows differentiation between benign and malignant nodules and significantly reduces unnecessary surgeries [5]. However, cytology has limitations, particularly in distinguishing follicular adenoma from carcinoma, where capsular and vascular invasion can only be demonstrated histologically [6]. Thus, histopathological examination remains the gold standard for final diagnosis and classification [7].

Radiological assessment, particularly high-resolution USG, is indispensable for initial evaluation, localization, and risk stratification of thyroid nodules. The American College of Radiology Thyroid Imaging Reporting and Data System (ACR TIRADS) and the American Thyroid Association (ATA) guidelines standardize sonographic interpretation and guide biopsy decisions [8,9]. Radiology complements cytology by identifying suspicious imaging features-such as hypoechogenicity, microcalcifications, and irregular margins correlate with an increased risk of malignancy [10].

Correlating histological, cytological, and radiological findings provides a comprehensive diagnostic approach that improves accuracy, minimizes false interpretations, and optimizes clinical management. Such integration ensures early diagnosis, appropriate surgical planning, and better prognostication in patients with thyroid lesions [7,10].

## Materials and methods

This cross-sectional, observational study was conducted in the Department of Pathology, Gandhi Medical College and Associated Hospitals, Bhopal, over a period of 18 months, from May 2023 to October 2024. The study commenced only after receiving approval from the Institutional Ethics Committee with approval number 18925/MC/IEC/2023 dated 09 May 2025. Patients presenting to various clinical departments with thyroid enlargement and referred for FNAC were recruited after obtaining written informed consent. All study procedures adhered to the ethical principles outlined in the Declaration of Helsinki.

Study population

The study included a total of 150 samples from patients with clinically palpable thyroid swellings. Each patient underwent thorough clinical evaluation, thyroid USG, thyroid function testing, and FNAC. Those who subsequently underwent thyroidectomy were further evaluated histopathologically to establish a definitive diagnosis and assess the correlation between cytological, radiological, and histological findings.

Inclusion and exclusion criteria

All patients presenting with thyroid swellings and referred for FNAC were included in the study. Patients with thyroid swellings associated with other primary malignancies, or those undergoing radiotherapy or chemotherapy, were excluded from the study. Thus, the study population consisted of consecutive patients with thyroid enlargement who met the inclusion criteria and provided consent to participate.

Sample size calculation and sampling method

The sample size was calculated using the formula:

\[ n = \frac{4pq}{d^2} \]

where *p* represents the prevalence of thyroid lesions from previous studies (12.2%) [11], *q* = 100 - *p* = 87.8, and *d* denotes the absolute error (12%). Based on these values, the minimum required sample size was 30 cases. However, to enhance statistical power, improve precision, and allow for subgroup analysis, a total of 150 subjects were included in the study. This larger sample size also accounted for potential data loss or incomplete records, ensuring robust and reliable results. A consecutive sampling method was employed, wherein all eligible patients meeting the inclusion criteria during the study period were consecutively enrolled until the target of 150 subjects was achieved.

Data collection

Detailed demographic, clinical, and laboratory data were collected using a structured proforma. The clinical information included the patient’s age, sex, and duration of symptoms, along with history regarding weight change, visual or mood alterations, menstrual irregularities, previous illnesses, radiation exposure, family history of thyroid disease, and use of hormonal therapy. General and local examination findings, such as the size, site, number, consistency, mobility, and tenderness of the thyroid swelling, were recorded. Associated symptoms like dysphagia, dyspnea, hoarseness of voice, palpitations, and heat or cold intolerance were also noted. These clinical observations were later correlated with cytological, radiological, and histopathological findings.

Investigations

USG Procedure

All patients underwent high-resolution USG of the thyroid gland using a linear transducer probe with a frequency range of 7.5-10 MHz. Each nodule was assessed for size, echogenicity, internal composition (solid, cystic, or mixed), margin regularity, presence of calcifications, and vascularity pattern. Based on these characteristics, the nodules were classified according to the TIRADS from category 1 to 5, where TIRADS 1 denoted a normal thyroid, TIRADS 2 benign lesions, TIRADS 3 low-suspicion nodules, TIRADS 4 intermediate-suspicion nodules, and TIRADS 5 high-suspicion nodules for malignancy. In cases with multiple nodules, the dominant or most suspicious nodule was chosen for correlation with cytological and histological findings.

Thyroid Function Tests

Approximately 2 mL of venous blood was drawn into a plain vial to separate serum for thyroid hormone estimation. The testing was carried out on a Beckman Coulter DxL800 (Beckman Coulter, Inc., Brea, CA) immunoassay analyzer. The reference ranges used for analysis were: T3, 0.8 to 2.0 ng/mL; T4, 5.1 to 14.1 µg/dL; and thyroid-stimulating hormone (TSH), 0.27 to 4.2 µIU/mL. Based on these values, patients were classified as euthyroid when all parameters were within normal limits, hypothyroid when T3 and T4 were reduced with elevated TSH levels, and hyperthyroid when T3 and T4 were elevated with low TSH levels.

Fine-Needle Aspiration Cytology 

FNAC was performed under aseptic precautions using a 23-gauge disposable needle attached to a 10 mL syringe, with or without the aid of a syringe holder. The swelling was localized, cleaned with an antiseptic solution, and aspirates were obtained from palpable lesions using either aspiration or non-aspiration techniques, depending on the consistency of the lesion. The aspirated material was expressed on clean glass slides and immediately smeared. Wet fixation in 95% ethanol was carried out for smears intended for Papanicolaou (PAP) and Hematoxylin and Eosin (H&E) staining, while air-dried smears were prepared for Giemsa staining.

For H&E staining, ethanol-fixed smears were stained in Harris hematoxylin for five minutes, washed in running tap water, differentiated in acid alcohol, blued in alkaline water, and counterstained with eosin for one minute, followed by dehydration, clearing, and mounting. For PAP staining, smears were fixed in 95% ethanol for fifteen to thirty minutes, stained with hematoxylin, and subsequently treated with OG-6 and EA-50 stains before dehydration and mounting. For Giemsa staining, air-dried smears were fixed in methanol for 3 minutes and stained with a freshly diluted Giemsa solution (1:10) for 20 minutes. They were then rinsed in buffered water and examined microscopically after drying. The cytological findings were categorized according to The Bethesda System for Reporting Thyroid Cytopathology (TBSRTC, 2017) into six categories: non-diagnostic or unsatisfactory (I), benign (II), atypia of undetermined significance or follicular lesion of undetermined significance (III), follicular neoplasm or suspicious for follicular neoplasm (IV), suspicious for malignancy (V), and malignant (VI).

Histopathology

Surgical management, including hemithyroidectomy, subtotal, near-total, or total thyroidectomy, was undertaken based on combined clinical, radiological, and cytological assessment. Excised thyroid specimens were fixed in 10% neutral buffered formalin for at least 24 hours. Gross examination included recording the size, shape, external surface, capsular integrity, color, and cut-surface features, such as cystic degeneration, hemorrhage, or necrosis. After fixation, representative sections were taken and processed routinely in a tissue processor. Paraffin blocks were prepared, and sections 3-5 µm thick were cut using a rotary microtome and mounted on glass slides. The sections were stained with H&E and examined microscopically. When required, Giemsa staining was used to highlight cytoplasmic details. The histological classification of thyroid lesions was carried out according to the World Health Organization (WHO, 2017) classification of thyroid tumors.

Observation parameters

For each patient, observations were made on age and sex distribution, duration of symptoms, site, size, and number of nodules, as well as other features such as consistency, mobility, and tenderness. The nature of aspirate, thyroid function status, clinical complaints, menstrual and past medical history, family history, radiation or hormonal exposure, cytological diagnosis (Bethesda category), radiological classification (TIRADS score), and histopathological diagnosis were recorded. Correlation was established between cytological and histopathological findings, as well as between TIRADS and cytology, and between TIRADS and histology, to determine diagnostic concordance.

Statistical analysis

All data were entered into Microsoft Excel and analyzed using IBM SPSS Statistics version 27.0 (IBM Corp., Armonk, NY). Descriptive statistics were applied to calculate frequencies and proportions for categorical variables, while continuous variables were expressed as mean ± standard deviation. The Chi-square test was used to assess associations between categorical variables. Correlations were evaluated between cytology and histopathology, TIRADS and cytology, and TIRADS and histopathology. A *P*-value <0.05 was considered statistically significant.

## Results

Characteristics of the study population

A total of 150 patients with thyroid lesions were analyzed. The majority were female (123, 82%), with a male-to-female ratio of approximately 1:4.6. The most common age group affected was 31-40 years (40, 26.7%), followed by 21-30 years (31, 20.7%) and 41-50 years (26, 17.3%).

Most lesions were diffuse (94, 62.7%), and more than half the patients (79, 52.7%) presented within one year of symptom onset. Among 122 female participants, 77 (63.1%) had regular menstrual cycles, 14 (11.5%) irregular, and 28 (23%) were postmenopausal. Common comorbidities included hypertension (6, 17.6%), tuberculosis (6, 17.6%), and hypothyroidism (5, 14.7%). Only 4 (2.7%) had a family history of thyroid disease, and 5 (3.3%) reported the use of exogenous hormones (Table 1).

**Table 1:** Characteristics of the study population (n = 150).

Parameter	Category	Frequency	Percentage (%)
Age group (years)	11-20	11	7.3
	21-30	31	20.7
	31-40	40	26.7
	41-50	26	17.3
	51-60	25	16.7
	> 60	17	11.3
Gender	Female	123	82.0
	Male	27	18.0
Site of lesion	Diffuse	94	62.7
	Left lobe	31	20.7
	Right lobe	25	16.7
Duration of symptoms (years)	0-1	79	52.7
	1.1-2	30	20.0
	2.1-3	10	6.7
	3.1-4	9	6.0
	4.1-5	7	4.7
	> 5	15	10.0
Menstrual pattern (*n* = 122)	Regular	77	63.1
	Irregular	14	11.5
	Postmenopausal	28	23.0
	Hysterectomy	3	2.5
Past medical history (*n* = 34)	Hypertension	6	17.6
	Tuberculosis	6	17.6
	Hypothyroidism	5	14.7
	Infection	5	14.7
	Graves’ disease	4	11.8
	Diabetes mellitus	2	5.9
	Hyperthyroidism	2	5.9
	Others (ear discharge, hyperlipidemia, anemia, neck surgery)	4	11.8
Family history of thyroid disease	Yes	4	2.7
	No	146	97.3
Use of exogenous hormones	Yes	5	3.3
	No	145	96.7

Clinical presentation

All patients presented with a neck swelling (150, 100%), the hallmark of thyroid lesions. Other frequent complaints included dysphagia (25, 16.7%), generalized weakness (23, 15.3%), weight loss (16, 10.7%), palpitation (15, 10%), and menstrual irregularities (14, 9.3%).

Most swellings were mobile (143, 95.3%) and non-tender (133, 88.7%). The predominant swelling size was 3-4 cm (38, 25.3%), followed by 2-3 cm (30, 20%) and 4-5 cm (25, 16.7%) (Table 2).

**Table 2:** Clinical features and size distribution (n = 150).

Parameter	Category	Frequency	Percentage (%)
Mobility	Mobile	143	95.3
	Fixed	7	4.7
Tenderness	Non-tender	133	88.7
	Tender	17	11.3
Size of swelling (cm)	0-1	2	1.3
	1-2	18	12.0
	2-3	30	20.0
	3-4	38	25.3
	4-5	25	16.7
	5-6	13	8.7
	6-7	12	8.0
	≥7	12	8.0

Cytological findings

FNAC was adequate in 144 (96%) cases. The most common aspirate was blood mixed with colloid (55, 36.7%), followed by scant blood mixed with colloid (52, 34.7%) and pure colloid (29, 19.3%) (Table 3).

**Table 3:** FNAC adequacy and aspirate characteristics (n = 150). FNAC, fine-needle aspiration cytology

Parameter	Category	Frequency	Percentage (%)
Diagnostic adequacy	Adequate	144	96.0
	Inadequate	6	4.0
Type of aspirate	Blood mixed colloid	55	36.7
	Scanty blood mixed	52	34.7
	Colloid	29	19.3
	Hemorrhagic	9	6.0
	Cyst fluid	5	3.3

On cytological examination, the most common diagnosis was colloid goiter (40, 26.7%), followed by lymphocytic thyroiditis (17, 11.3%), colloid cyst (15, 10%), and nodular goiter (15, 10%). Malignant lesions comprised 5 cases (3.3%) overall. According to the Bethesda system, 123 cases (82%) were benign (Category II), 10 (6.7%) were non-diagnostic (Category I), and 4 (2.7%) were malignant (Category VI) (Table 4).

**Table 4:** Cytological diagnosis and Bethesda classification (n = 150). FLUS, Follicular Lesion of Undetermined Significance

Bethesda category	Frequency	Percentage (%)
I - Non-diagnostic	10	6.7
II - Benign	123	82.0
III - Atypia/FLUS	3	2.0
IV - Follicular neoplasm	8	5.3
V - Suspicious for malignancy	2	1.3
VI - Malignant	4	2.7
Total	150	100

Thyroid function test correlation

**Figure 1:**
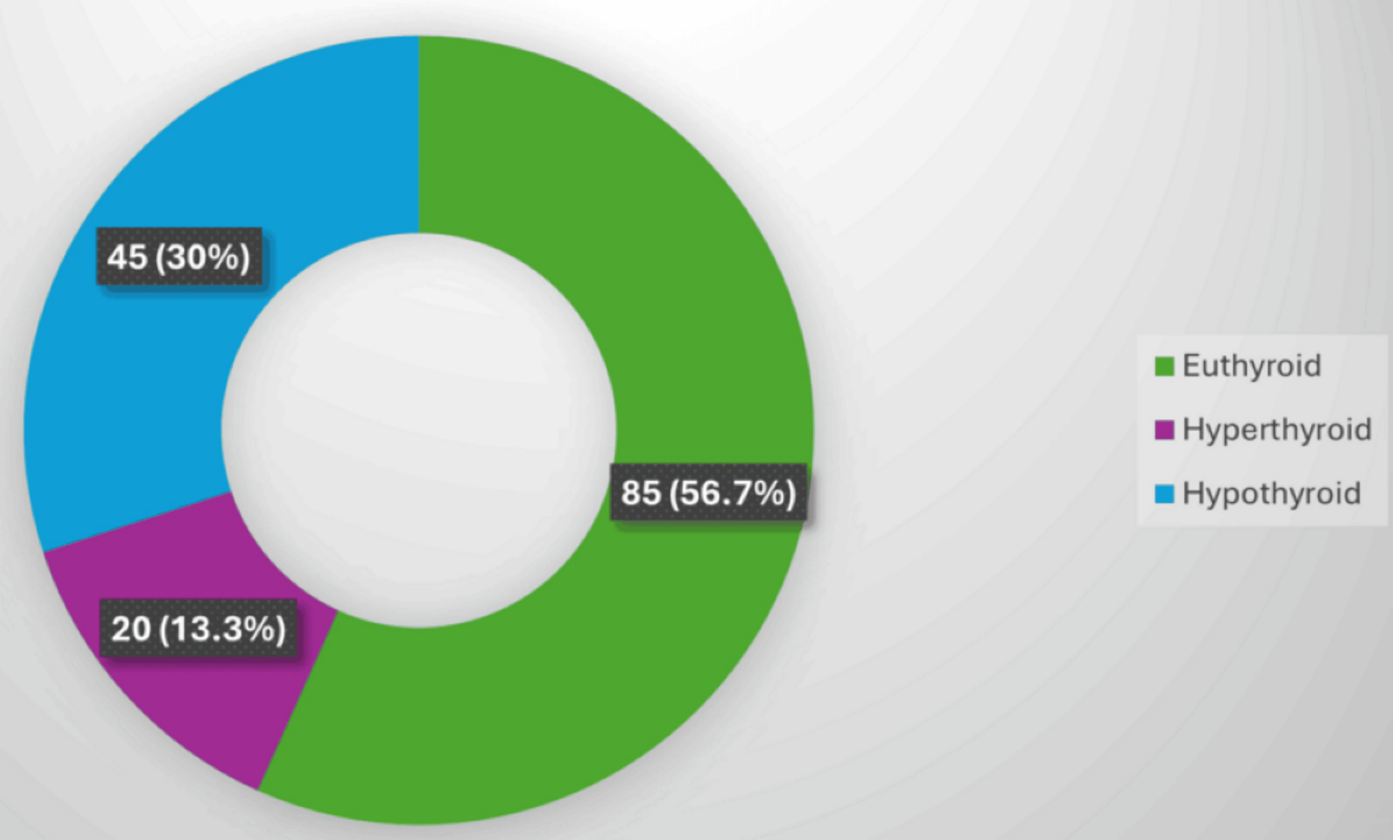
Thyroid function test results (n = 150).

Thyroid function testing revealed that 85 cases (56.7%) were euthyroid, 45 (30%) were hypothyroid, and 20 (13.3%) were hyperthyroid. Colloid goiter predominated among individuals who were euthyroid (25, 29.4%), lymphocytic thyroiditis among those with hypothyroidism (12, 26.7%), and hyperplastic nodules among those with hyperthyroidism (6, 30%) (Figure 1).

Radiological (TIRADS) findings

Using the ACR TIRADS system, most nodules were TIRADS 3 (63, 42%) and TIRADS 2 (58, 38.7%), while TIRADS 4 (18, 12%) and TIRADS 5 (7, 4.7%) represented higher-risk categories. A significant association existed between higher TIRADS scores and cytological malignancy (*P* < 0.001) (Table 5).

**Table 5:** TIRADS classification (n = 150). TIRADS, Thyroid Imaging Reporting and Data System

TIRADS category	Frequency	Percentage (%)
1	4	2.7
2	58	38.7
3	63	42.0
4	18	12.0
5	7	4.7
Total	150	100.0

Surgical and histopathological findings

Among 52 patients who underwent surgery, hemithyroidectomy (23, 44.2%) was the most frequent procedure, followed by total thyroidectomy (17, 32.7%) and subtotal thyroidectomy (9, 17.3%) (Table 6).

**Table 6:** Distribution of patients according to the type of surgery (n = 52).

Procedure	Frequency	Percentage (%)
Hemithyroidectomy	23	44.2
Subtotal thyroidectomy	9	17.3
Total thyroidectomy	17	32.7
Near-total thyroidectomy	1	1.9
Others	2	3.9
Total	52	100

Histopathology revealed thyroid follicular nodular disease (21, 40.4%) as the most common lesion, followed by follicular adenoma (6, 11.5%), simple goiter (4, 7.7%), and papillary carcinoma (4, 7.7%). Overall, malignant cases comprised 17.3% (Table 7).

**Table 7:** Distribution of patients as per histopathological diagnosis (n = 52). NIFTP, noninvasive follicular thyroid neoplasm with papillary-like nuclear features

Lesion	Frequency	Percentage (%)
Follicular nodular disease	21	40.4
Follicular adenoma	6	11.5
Simple goiter	4	7.7
Papillary carcinoma	4	7.7
Medullary carcinoma	2	3.8
Anaplastic carcinoma	2	3.8
NIFTP	2	3.8
Lymphocytic thyroiditis	3	5.8
Others (e.g., oncocytic adenoma, Graves' disease, etc.)	6	11.5
Total	52	100.0

Diagnostic correlation and accuracy

Comparing cytology with histopathology, FNAC demonstrated a sensitivity of 62.5%, a specificity of 97.7%, and an overall concordance of 92.3%. All TIRADS 5 lesions proved malignant on histology; the Bethesda VI category had complete confirmation (100%) (Table 8).

**Table 8:** Diagnostic correlation and performance indices (n = 52). Values are expressed as percentages. *P*-values were derived using the chi-square (χ²) test to determine the statistical association between diagnostic modalities and histopathological findings. A *P*-value < 0.05 was considered statistically significant. FNAC, fine-needle aspiration cytology; TIRADS, Thyroid Imaging Reporting and Data System

Parameter	Value	Test statistics (*χ*²)
FNAC sensitivity	62.5%	12.46
FNAC specificity	97.7%	28.12
Cytology–histology concordance	92.3%	31.85
TIRADS–histology correlation	*P* < 0.001 (significant)	34.29

Four discordant cases were identified: two papillary carcinomas, one medullary carcinoma, and one case of Graves' disease misinterpreted cytologically.

## Discussion

Thyroid lesions represent a broad spectrum of pathologies ranging from non-neoplastic to malignant neoplasms, with a higher prevalence among women in the third and fourth decades of life. In the present study, the majority of cases were benign, with colloid goiter being the most common lesion on both cytology and histopathology. These findings are consistent with those reported by Rani et al. [12], who observed that benign lesions constituted 81.6% of thyroid swellings, and colloid goiter was the predominant diagnosis with a cytological accuracy of 93.8%. Similarly, Yadav et al. [13] reported a predominance of Bethesda II (benign) lesions (76%) among euthyroid individuals, reinforcing the relationship between functional status and benign cytology. In contrast, Abdullah et al. [14] and Kamboj et al. [15] demonstrated a higher proportion of malignancy, 14% and 34.6%, respectively, likely reflecting regional variations and patient selection differences.

The thyroid function profile in our series revealed that 56.7% of patients were euthyroid, followed by 30% hypothyroid and 13.3% hyperthyroid, suggesting that most thyroid nodules occur in a euthyroid state. These findings align with those of Kudva and Kishore [16] and Almahari et al. [17], who reported that structural abnormalities in euthyroid individuals are predominantly benign and often associated with colloid degeneration or autoimmune thyroiditis. Among hypothyroid patients, lymphocytic thyroiditis was the most frequent lesion (26.7%), aligning with the known autoimmune etiology of hypothyroidism. This finding was comparable to those of Kudva and Kishore [16], who emphasized cytomorphological changes consistent with Hashimoto’s thyroiditis in hypothyroid states. In hyperthyroid patients, hyperplastic nodules (30%) predominated, consistent with Paschke et al. [18], who described toxic multinodular goitre as a major cause of hyperthyroidism in iodine-sufficient regions.

Radiologically, TIRADS proved to be a valuable non-invasive tool in risk stratification. In our study, the majority of nodules were classified as TIRADS 2 and 3, which correlated with benign cytology. In contrast, all TIRADS 5 nodules were found to be malignant on histopathology, confirming a strong association between the TIRADS category and the risk of malignancy (p < 0.001). This observation closely parallels the results of Al-Chalabi et al. [19], who validated the British Thyroid Association ultrasound classification and found a significant correlation between higher TIRADS grades and malignant potential. Likewise, Biswas et al. [20] observed a direct relationship between increasing TIRADS category and Bethesda cytological class, concluding that TIRADS could effectively guide the need for FNAC.

FNAC remains the cornerstone of thyroid evaluation due to its simplicity, cost-effectiveness, and high diagnostic reliability. In our study, the cytological adequacy rate was 96%, and the cyto-histological concordance rate was 92.3%, with a sensitivity of 62.5%, a specificity of 97.7%, and an overall diagnostic accuracy of 92.31%. These values are in close agreement with Roy et al. [21] and Bhadouria et al. [22], who reported sensitivities ranging from 60% to 70% and specificities exceeding 90%. Similarly, Jamaiyar and Yogesh [23] observed an overall diagnostic accuracy of 93%, affirming FNAC as a highly dependable first-line modality for thyroid nodules. Discordance between cytology and histology in our series (7.6%) was primarily due to sampling error, where focal malignancies were missed during aspiration - a limitation also highlighted by Bamanikar et al. [24] and Warpe et al. [25].

The cytoradiological correlation in our study further strengthened the combined diagnostic value of FNAC and TIRADS. As noted by Raniwala et al. [26] and Warpe et al. [25], integrating ultrasonographic features with cytology enhances preoperative diagnostic confidence, reduces unnecessary surgeries, and ensures early detection of malignancies. Moreover, studies by Singh et al. [27] and Osseis et al. [28] have advocated for a multidisciplinary approach that combines clinical, radiological, cytological, and histopathological data for accurate diagnosis of thyroid lesions.

The present study had certain limitations. Being a single-center, hospital-based study, the sample size was relatively limited and may not represent the entire population spectrum of thyroid lesions. A few cases showed discordance between cytological and histopathological findings, likely due to sampling error or the heterogeneous nature of large nodules, where focal malignant areas might have been missed during aspiration. The study did not incorporate ancillary techniques such as immunocytochemistry or molecular testing, which could have improved diagnostic accuracy in indeterminate cases. Additionally, long-term follow-up of patients was not undertaken to assess disease recurrence or progression. Future multicentric studies with larger cohorts and the inclusion of advanced diagnostic modalities are recommended to validate and strengthen these findings.

## Conclusions

The present cross-sectional study highlighted that the majority of thyroid lesions are benign, with colloid goiter being the most common finding, predominantly affecting middle-aged females with diffuse and euthyroid swellings. FNAC has proven to be a simple, cost-effective, and highly reliable diagnostic tool, demonstrating strong concordance with histopathological findings. USG, classified according to the TIRADS, effectively stratified the risk of malignancy, and its correlation with FNAC and histopathology enhanced diagnostic precision. The integration of radiological, cytological, and histopathological assessments thus provided a comprehensive approach for accurate diagnosis, rational surgical decision-making, and improved patient management in thyroid lesions.
